# Crystal and Electronic Structures, Photoluminescence Properties of Eu^2+^-Doped Novel Oxynitride Ba_4_Si_6_O_16-3x/2_N_x_

**DOI:** 10.3390/ma3031692

**Published:** 2010-03-08

**Authors:** Yuanqiang Li, Yuan Fang, Naoto Hirosaki, Rong-Jun Xie, Lihong Liu, Takashi Takeda, Xiaoyun Li

**Affiliations:** 1 College of Materials Science and Engineering, Nanjing University of Technology, New Model Road 5, Nanjing, Jiangsu 210009, China; E-Mails: fy851215@126.com (Y.F.); lixiaoyun@njut.edu.cn (X.L.); 2 Nitride Particle Group, Nano Ceramics Center, National Institute for Materials Science, Namiki 1-1, Tsukuba, Ibaraki 305-0044, Japan; E-Mails: hirosaki.naoto@nims.go.jp (N.H.); xie.rong-jun@nims.go.jp (R.J.X.); liu.lihong@nims.go.jp (L.L.); takeda.takashi@nims.go.jp (T.T.)

**Keywords:** barium silicon oxynitride, europium, crystal structure, electronic structure, luminescence properties, thermal quenching

## Abstract

The crystal structure and the photoluminescence properties of novel green Ba_4-y_Eu_y_Si_6_O_16-3x/2_N_x_ phosphors were investigated. The electronic structures of the Ba_4_Si_6_O_16_ host were calculated by first principles pseudopotential method based on density functional theory. The results reveal that the top of the valence bands are dominated by O-2p states hybridized with Ba-6s and Si-3p states, while the conduction bands are mainly determined by Ba-6s states for the host, which is an insulator with a direct energy gap of 4.6 eV at Γ. A small amount of nitrogen can be incorporated into the host to replace oxygen and forms Ba_4-y_Eu_y_Si_6_O_16-3x/2_N_x_ solid solutions crystallized in a monoclinic (space group *P*2_1_/c, Z = 2) having the lattice parameters *a* = 12.4663(5) Å, *b* = 4.6829(2) Å, *c* = 13.9236(6) Å, and *β* = 93.61(1)°, with a maximum solubility of nitrogen at about x = 0.1. Ba_4_Si_6_O_16-3x/2_N_x_:Eu^2+^ exhibits efficient green emission centered at 515–525 nm varying with the Eu^2+^ concentration when excited under UV to 400 nm. Furthermore, the incorporation of nitrogen can slightly enhance the photoluminescence intensity. Excitation in the UV-blue spectral range (λ_exc_ = 375 nm), the absorption and quantum efficiency of Ba_4-y_Eu_y_Si_6_O_16-3x/2_N_x_ (x = 0.1, y = 0.2) reach about 80% and 46%, respectively. Through further improvement of the thermal stability, novel green phosphor of Ba_4-y_Eu_y_Si_6_O_16-3x/2_N_x_ is promising for application in white UV-LEDs.

## 1. Introduction

Recently, in the exploration of novel phosphors for applications in white LED lighting, several oxynitride based phosphors with improved properties have been created by partial cross-substitution of Si-N for Al-O in the oxide based host lattices, like alkaline earth aluminates of MAl_2_O_4_:Eu^2+^ (M = Ca, Sr, Ba) [1] and alkaline earth aluminosilicates, viz., Sr_2_Al_2_SiO_7_:Eu^2+^ [2] and Sr_3_Al_10_SiO_20_:Eu^2+^ [3,4]. By this approach, the absorption and excitation bands of Eu^2+^ can be extended to the longer wavelength [1,2] and the emission intensity can be enhanced [3,4] due to the incorporation of more covalent bond of Si-N, providing a stronger reducing environment around the Eu^2+^ ions in the oxide based host lattices [5]. On the other hand, it is also highly interesting to know whether or not a single nitrogen can be incorporated into the oxide based lattices occupied on the oxygen sites, which could be more flexible than the cross-substitution of Si-N ➔ Al-O and increase the possibility of inventing novel oxynitride phosphor materials to meet the requirements of the development of white LEDs.

The luminescence properties of rare-earth doped alkaline-earth silicates have been widely investigated [6,7,8,9,10,11,12,13,14,15,16]. Particularly, barium containing compounds are of great interest for practical luminescent materials due to higher luminous efficiency of Eu^2+^ in this kind of host lattices. Under UV excitation, Eu^2+^-doped barium silicates can be roughly classified into two groups according to the emitting color: bluish-green emission, *i.e.*, Ba_2_SiO_4_:Eu^2+^ (λ_em_ = 504–510 nm) [6,7,8,9,10,11,12,13], BaSi_2_O_5_:Eu^2+^ (λ_em_ ~ 520 nm) [6,7], and Ba_2_Si_3_O_8_:Eu^2+^ (~485 nm) [7,8]; and greenish-yellow emission, *i.e.*, BaSiO_3_:Eu^2+^ (~550 nm) [7] and Ba_3_SiO_5_:Eu^2+^ (504–566 nm or 560*–*590 nm) [14, 15]. The position of the emission band mainly depends on the chemical composition and crystal structure. In general, the bond length of Ba-O ranges from 2.62 to 3.06 Å with the coordination numbers varying from 7 to 10 in barium silicates [17]. As a pilot study, barium silicates have been selected as the mother host based on the fact that the average bond length is longer than that of Ca-O and Sr-O due to their large ionic radius of Ba^2+^ in ternary alkaline earth silicates, which means the bonding of Ba-O is relatively weaker that enables nitrogen more easily to replace oxygen in barium silicates.

As far as we know, no related such reports have been found, and the correlations of the crystal and electronic structure with the luminescence properties also have not yet been studied in the barium silicate system of Ba_4_Si_6_O_16_. As one of our exploration investigations, in the present work Ba_4_Si_6_O_16_ was used as the host lattice to check the possibility of the incorporation of single nitrogen by the general formula of Ba_4-y_Eu_y_Si_6_O_16-3x/2_N_x_. For a better understanding of the photoluminescence properties, firstly we calculated the electronic structures of the host lattice of Ba_4_Si_6_O_16_ by first principles method, then we characterized the crystal structure, photoluminescence properties, and thermal stability of Eu^2+^-doped Ba_4_Si_6_O_16-3x/2_N_x_.

## 2. Computational Details

The density of states (DOS) and band structure calculation for Ba_4_Si_6_O_16_ were performed by first principles method using pseudopotentials and a plane wave basis set [18] within the density functional theory (DFT) performed by VASP package [19,20,21]. The initial structural parameters were adopted from the single crystal data [22]. The projector augmented wave pseudopotentials were adapted for Ba, Si and O atoms. Exchange correlations were treated with the generalized gradient approximation (GGA) with a Perdew-91 functional form [23]. The numerical integration of the Brillouin zone (BZ) was performed using a discrete 4 × 6 × 4 Monkhorst-Pack *k*-point sampling, and the plane wave cutoff energy was fixed at 500 eV. The Wigner-Seitz radius employed in the calculations is about 1.979 Å for Ba, 1.312 Å for Si, and 0.82 Å for O. The Fermi energy level was set at zero energy for the calculations.

## 3. Experimental Section

### 3.1. Synthetic approaches

The oxynitride phosphors with the composition of Ba_4-y_Eu_y_Si_6_O_16-3x/2_N_x_ (x < 1, and y = 0.04*–*0.4) were synthesized by a solid state reaction approach using BaCO_3_ (Sigma-Aldrich, 99%), SiO_2_ (High Purity Chemical Co., Ltd, 99.9%), Si_3_N_4_ (UBE, SN-E10), and Eu_2_O_3_ (Shin-Etsu Chemical Co. Ltd., 99.99%) as the starting materials. The appropriate amount of the raw materials were weighted out and then mixed by ball milling in hexane with silicon nitride balls for about 4 h. Subsequently, the dried powder mixtures were diverted in a BN boat and then fired within a tube furnace at 1200*–*1300 °C for 6 h under a N_2_-H_2_ (5%) atmosphere.

### 3.2. Characterization

The X-ray diffraction (XRD) patterns of the prepared materials were recorded by the X-ray powder diffraction (Rigaku, RINT Ultima-III) with the graphite monochromator using Cu-K_α_ radiation (λ = 1.54056 Å), operating at 40 kV and 40 mA. For the structure analysis, the XRD data were collected in the range of 10–100° in 2*θ* using a step-scan mode with a step size of 0.02 and a count time of 5 s per step. The Rietveld refinements were carried out by the GSAS package [24,25]. The structural parameters of Ba_4_Si_6_O_16_ [22] were used as an initial model for the refinement of the crystal structure of Ba_4-y_Eu_y_Si_6_O_16-3x/2_N_x_, and the Eu and N ions are supposed to be randomly occupied on Ba and O sites, respectively, in the course of structural refinements.

The photoluminescence spectra were measured by a fluorescent spectrophotometer (F-4500, Hitachi Ltd., Japan) at room temperature with a 150 W xenon short arc lamp. The emission spectrum was corrected for the spectral response of a monochrometer and Hamamatsu R928P photomultiplier tube by a light diffuser and tungsten lamp. The excitation spectrum was also corrected for the spectral distribution of the xenon lamp intensity by measuring rhodamine-B as the reference. The quantum efficiency and the temperature-dependent luminescence properties were recorded on an intensified multichannel spectrophotometer (MCPD-7000, Otsuka Electrics, Japan) with a 200 W Xe lamp as an excitation source. A white BaSO_4_ plate was employed as a standard reference for the quantum efficiency measurement. With regard to the thermal stability measurement, the powder samples within a quartz container were heated from room temperature to 200 °C in air with a heating rate at 100 °C/min, and the time duration was set for 5 min at each recorded temperature.

## 4. Results and Discussion

### 4.1. Electronic structures of the Ba_4_Si_6_O_16_ host

**Figure 1:**
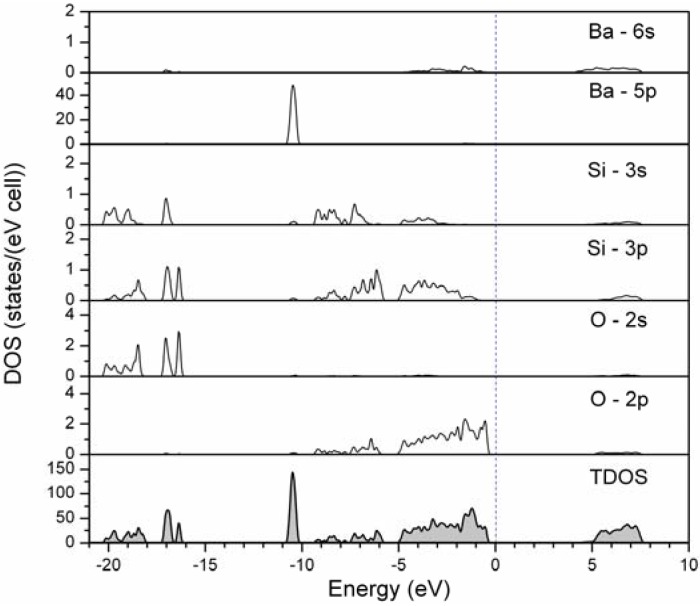
Partial and total density states (DOS) for the host of Ba_4_Si_6_O_16_.

Figure 1 shows the partial density states (DOS) for the Ba, Si and O atoms, as well as total density of states for the host of Ba_4_Si_6_O_16_. It can be seen that the valence band is mainly composed of the valence electrons of Ba-6s, Si-3s3p, and O-2p states with a bandwidth of about 9.5 eV. The Ba-5p states have the character of semicore level states with an intense peak centered at about −10.5 eV, far below the valence bands, implying weak interaction between the Ba-5p and O-2s2p states due to relatively long average Ba-O distances (2.791 and 2.851 Å for Ba1-O and Ba2-O, respectively). The O-2p states are almost fully occupied within the valence band (−9.5*–*0 eV), while the Ba-6s state is significantly weaker, Ba-O can be regarded as ionic bonding. The lower part of the valence band ranging from −16 to*–*20 eV consists of several sharp narrow bands, which are composited of the O-2s states hybridized with partial Si-3s3p states as well as a little Ba-6s states (Figure 1). The top of the valence band is dominated by the O-2p states hybridized with the Ba-6s and Si-3p states that contribute to the chemical bonding in the Ba_4_Si_6_O_16_ compound, having some covalent characters. Moreover, Ba-O also contains some covalent character in its predominated ionic bonding. The bottom of the conduction band of Ba_4_Si_6_O_16_ is dominated by Ba-6s states at around 4.05 eV, while the other states of Ba-5p, Si-3s3p and O-2s2p constituted the valence bands are distributed at higher energy between 5–7.8 eV.

**Figure 2:**
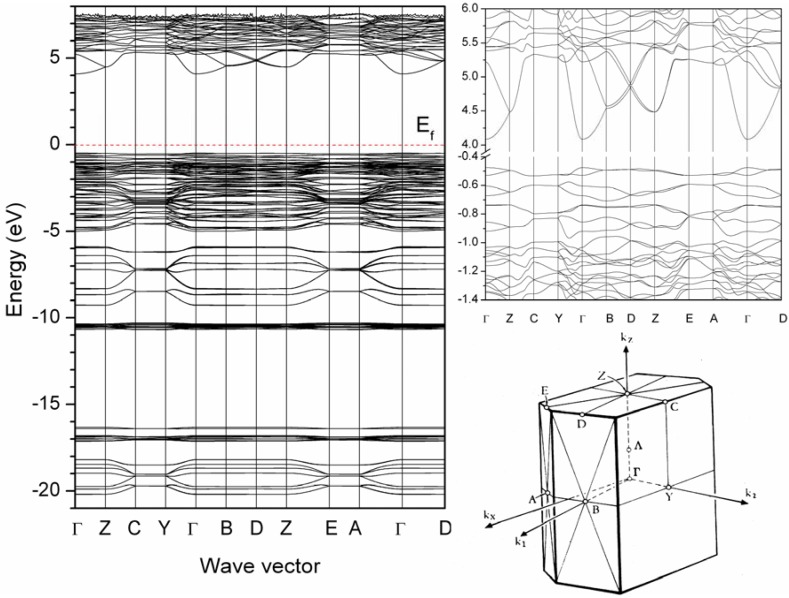
Band structure of Ba_4_Si_6_O_16_ and the high symmetry points in the Brillouin zone of the Ba_4_Si_6_O_16_ lattice.

Figure 2 illustrates the calculated distribution curves for the band structure of Ba_4_Si_6_O_16_ along the high symmetry lines. Corresponding to the DOS as mentioned above (see Figure 1), the bottom of the conduction band with the Ba-6s states is located at the Γ point in the Brillouin zone (BZ), and the top of the valence band with O-2p states is also at the Γ point (see an enlarged figure in Figure 2). Therefore, Ba_4_Si_6_O_16_ is an insulator material with a direct energy gap of about 4.6 eV. With such a large band gap, it is expected that the energy levels of the 4f^6^5d ↔ 4f^7^ transitions of the Eu^2+^ ion in the host lattice of Ba_4_Si_6_O_16_ should have small interferences with the valence and conduction bands.

### 4.2.Formation and crystal structure of Ba_4-y_Eu_y_Si_6_O_16-3x/2_N_x_

**Figure 3:**
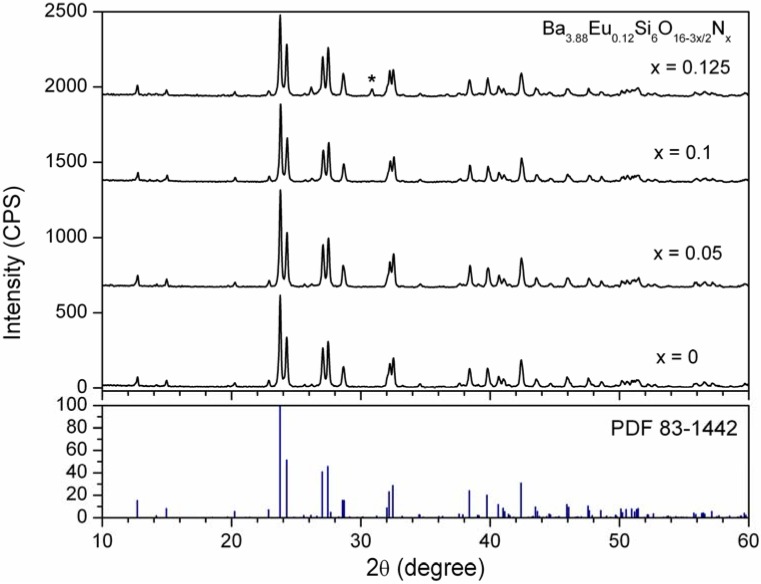
X-ray powder diffraction of Ba_3.88_Eu_0.12_Si_6_O_16-3x/2_N_x_(x = 0*–*0.125) along with a reference pattern of PDF 83-1442 for Ba_4_Si_6_O_16_.

Figure 3 shows the X-ray powder diffraction patterns of Ba_3.88_Eu_0.12_Si_6_O_16-3x/2_N_x_. It was evidently found that single nitrogen could be incorporated into the host lattice of Ba_4_Si_6_O_16_ through partial replacement of the oxygen atoms to form the limited solid solutions in a single phase form. The maximum solubility of nitrogen is only about x = 0.1 based on the fact that the Ba_3.88_Eu_0.12_Si_6_O_16-3x/2_N_x_ phosphors are single phase products at x ≤ 0.1, nevertheless an unindexed second-phase occurs when the x value surpasses 0.1. The O/N ratio measured by chemical analysis was also given the similar result (16/0.12). As compared to the cross-substitution of Si-N ➔ Al-O, the lower solubility of nitrogen within metal silicates could be mainly related to the type of the crystal structure, as well as the composition, e.g., the Ba/Si ratio.

**Figure 4:**
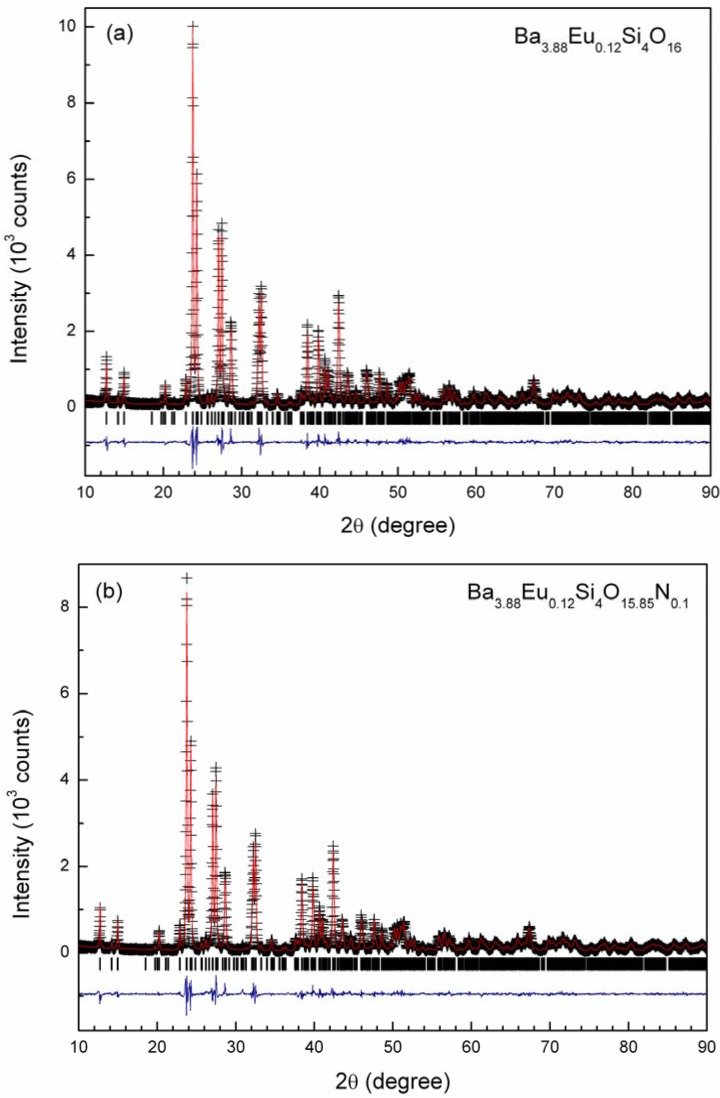
Rietveld refinement of X-ray diffraction patterns for Ba_3.88_Eu_0.12_Si_6_O_16-3x/2_N_x_, (a) x = 0 and (b) x = 0.1. The observed counts are indicated by crosses and the calculated pattern by a solid line. The residual curve is shown in the bottom of the diagram. The vertical bars indicate the position of Bragg reflections.

**Figure 5:**
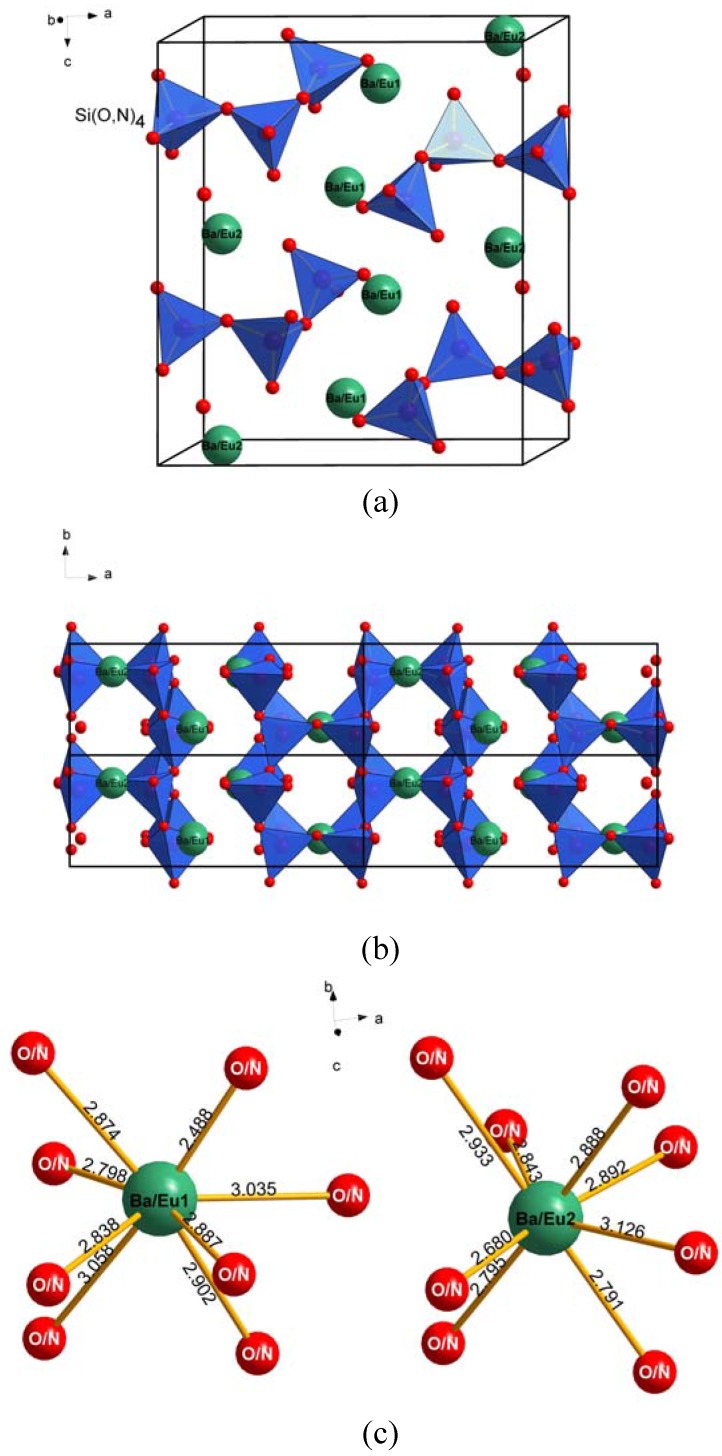
Projection of the crystal structure of Ba_3.88_Eu_0.12_Si_6_O_16-3x/2_N_x_ (x = 0.1) viewed along (a) (010), (b) (001), and (c) the local coordination of the Ba/Eu atoms with O/N atoms. The large green sphere represents Ba/Eu atoms and small red sphere represents O/N atoms.

Figure 4 depicts the Rietveld refinement of X-ray powder diffraction patterns of Ba_3.88_Eu_0.12_Si_6_O_16-3x/2_N_x_ for x = 0 and 0.1. As shown in Figure 4, the observed XRD pattern perfectly matches with that of the calculated one, confirming that small amounts of the nitrogen atoms can partially replace the oxygen atoms in the Ba_4_Si_6_O_16_ host lattice and form the defected (*i.e.*, oxygen vacancy) solid solution of Ba_3.88_Eu_0.12_Si_6_O_16-3x/2_N_x_. The perspective view of the crystal structure of Ba_3.88_Eu_0.12_Si_6_O_16-3x/2_N_x_ is shown in Figure 5 along with the local coordination of the Ba/Eu atoms with the O/N atoms (Figure 5c).

In comparison with oxide based Ba_3.88_Eu_0.12_Si_6_O_16_, the lattice parameters of Ba_3.88_Eu_0.12_Si_6_O_16-3x/2_N_x_ (x = 0.1) are slightly expanded by introducing the nitrogen atom because the ionic size of N^3-^ (1.46 Å) is larger than that of O^2-^ (1.38 Å) in the four-fold coordination [26]. As a consequence, both the unit cell volume and the average bond distance of Ba/Eu-O/N show slight increase for the obtained oxynitride phosphor due to the size effect. In addition, the unit cell volumes of Eu^2+^-doped Ba_4_Si_6_O_16_ and Ba_4_Si_6_O_16-3x/2_N_x_ (x = 0.1) also show small shrinkage as compared to a single crystal Ba_4_Si_6_O_16_, *i.e.*, V = 813.54 Å^3^ [22], in agreement with the factor that Eu^2+^ (1.25 Å for CN = 8) is smaller that that of Ba^2+^ (1.42 Å for CN = 8) [26]. The crystallographic data and selected bond distances of Ba_3.88_Eu_0.12_Si_6_O_16-3x/2_N_x_ (x = 0 and 0.1) are summarized in Table 1, in which the derived structural data of Eu^2+^-doped Ba_4_Si_6_O_16_ oxide based compound are comparable to those determined in the previous studies [22].

Table 1Crystallographic data, selected bond distances (Å) and the coordination polyhedron volume (Å^3^) around Ba_Eu_ for Ba_3.88_Eu_0.12_Si_6_O_16-3x/2_N_x_, (a) x = 0, and (b) x = 0.1.

**Table:** (a) x = 0

Formula				Ba_3.88_Eu_0.12_Si_6_O_16_						
Formula weight				975.57						
Crystal system				Monoclinic						
Space group				*P*2_1_/c (14)						
*Z*				2						
Lattice parameters										
a (Å)				12.4651(4)						
b (Å)				4.6826(1)						
c (Å)				13.9242(4)						
*β* (°)				93.60(1)						
Unit cell volume (Å^3^)				811.13(4)						
Density (g•cm^-3^)										
ρ_calc._				3.994						
*R_wp_*				9.6%						
*R_p_*				7.3%						
χ^2^				2.78						
Atom	Wyck.	*x/a*			*y/b*		*z/c*	S.O.F.		*U* (100Å^2^)
Ba/Eu1	4e	0.5806(23)			0.7673(11)		0.1123(17)	0.97/0.03		1.67
Ba/Eu2	4e	0.8548(23)			0.2523(12)		0.0329(18)	0.97/0.03		1.53
Si1	4e	0.6475			0.8026		0.3892	1.00		2.23
Si2	4e	0.7240			0.3230		0.2761	1.00		1.96
Si3	4e	0.9717(9)			0.3201(33)		0.3054(8)	1.00		1.42
O1	4e	0.5282(15)			0.7620(11)		0.4091(14)	1.00		1.62
O2	4e	0.7410(14)			0.7810(8)		0.4759(13)	1.00		0.88
O3	4e	0.6445			0.1494		0.3486	1.00		2.62
O4	4e	0.6805(19)			0.639(5)		0.2970(16)	1.00		1.04
O5	4e	0.7151			0.2375		0.1692	1.00		1.29
O6	4e	0.8409(16)			0.2220(8)		0.3255(13)	1.00		1.64
O7	4e	0.0373(14)			0.2430(10)		0.4072(12)	1.00		2.55
O8	4e	-0.0053(17)			0.6510(6)		0.2826(19)	1.00		1.76
**(Ba/Eu)1-O**						**(Ba/Eu)2-O**				
Ba/Eu1-O1			2.730(5)			Ba/Eu2-O2			2.96(4)	
Ba/Eu1-O1			2.690(4)			Ba/Eu2-O2			2.697(32)	
Ba/Eu1-O1			2.866(19)			Ba/Eu2-O5			2.655(3)	
Ba/Eu1-O2			2.851(17)			Ba/Eu2-O6			2.885(18)	
Ba/Eu1-O3			2.943(3)			Ba/Eu2-O7			2.840(4)	
Ba/Eu1-O4			2.852(19)			Ba/Eu2-O7			2.770(4)	
Ba/Eu1-O5			3.070(4)			Ba/Eu2-O7			2.957(17)	
Ba/Eu1-O5			2.850(5)			Ba/Eu2-O8			3.120(21)	
mean			2.857 ± 0.117			mean			2.860 ± 0.154	
polyhedron volume			97.21			polyhedron volume			96.88

**Table:** (b) x = 0.1

Formula				Ba_3.88_Eu_0.12_Si_6_O_15.85_N_0.1_						
Formula weight				974.57						
Crystal system				Monoclinic						
Space group				*P*2_1_/c (14)						
*Z*				4						
Lattice parameters										
a (Å)				12.4663(5)						
b (Å)				4.6829(1)						
c (Å)				13.9236(6)						
*β* (°)				93.61(1)						
Unit cell volume (Å^3^)				811.22(6)						
Density (g•cm^-3^)										
ρ_calc._				3.990						
*R_wp_*				9.8%						
*R_p_*				7.3%						
χ^2^				2.48						
Atom	Wyck.	*x/a*			*y/b*		*z/c*	S.O.F.		*U* (100Å^2^)
Ba/Eu1	4e	0.5802(2)			0.7629(13)		0.1124(2)	0.97/0.03		1.61
Ba/Eu2	4e	0.8556(2)			0.2515(13)		0.0319(2)	0.97/0.03		1.41
Si1	4e	0.6494			0.7871		0.3915	1.00		2.05
Si2	4e	0.7241			0.3220		0.2759	1.00		1.81
Si3	4e	0.9715			0.3202		0.3052	1.00		1.33
O/N1	4e	0.5254(15)			0.7120(8)		0.4080(14)	0.9906/0.0063		1.68
O/N2	4e	0.7366			0.7665		0.4732	0.9906/0.0063		1.80
O/N3	4e	0.6495(17)			0.1220(5)		0.3446(14)	0.9906/0.0063		2.12
O/N4	4e	0.6777(20)			0.6460(5)		0.2947(15)	0.9906/0.0063		1.53
O/N5	4e	0.7153			0.2379		0.1700	0.9906/0.0063		1.23
O/N6	4e	0.8416(16)			0.2490(11)		0.3273(11)	0.9906/0.0063		1.27
O/N7	4e	0.0314(14)			0.2390(11)		0.4060(11)	0.9906/0.0063		2.48
O/N8	4e	-0.0054			0.6488		0.2827	0.9906/0.0063		1.56
**(Ba/Eu)1-O/N**						**(Ba/Eu)2-O/N**				
Ba/Eu1-O/N1			2.900(4)			Ba/Eu2-O/N2			2.933(5)	
Ba/Eu1-O/N1			2.490(34)			Ba/Eu2-O/N2			2.795(5)	
Ba/Eu1-O/N1			2.887(20)			Ba/Eu2-O/N5			2.680(3)	
Ba/Eu1-O/N2			2.837(3)			Ba/Eu2-O/N6			2.844(16)	
Ba/Eu1-O/N3			3.035(21)			Ba/Eu2-O/N7			2.890(4)	
Ba/Eu1-O/N4			2.798(19)			Ba/Eu2-O/N7			2.790(4)	
Ba/Eu1-O/N5			3.058(5)			Ba/Eu2-O/N7			2.892(16)	
Ba/Eu1-O/N5			2.873(5)			Ba/Eu2-O/N8			3.126(2)	
mean			2.860 ± 0.175			mean			2.868 ± 0.130	
polyhedron volume			97.22			polyhedron volume			98.03	

Ba_3.88_Eu_0.12_Si_6_O_16-3x/2_N_x_ (x = 0.1) is isostructural with Ba_4_Si_6_O_16_ [22] crystallized in a monoclinic system with the space group P2_1_/c (No. 14), having the lattice parameters *a* = 12.4663(5) Å, *b* = 4.6829(2) Å, *c* = 13.9236(6) Å, and *β* = 93.61(1)°, *Z* = 2. In Ba_3.88_Eu_0.12_Si_6_O_16-3x/2_N_x_, the [Si(O,N)]_4_ tetrahedra are corner-sharing to form two single chains, namely sub-chains, and three such single chains are linked into triple chains running along [010] with 2_1_ symmetry [22]. Two individual Ba/Eu atoms are located in between two triple chains for the (Ba/Eu)1 and between two single chains for (Ba/Eu)2 atoms (Figure 5c). Both (Ba/Eu)1 and (Ba/Eu)2 are directly coordinated by eight O/N atoms and build up the [Ba_Eu_(O,N)_8_] polyhedra (Figure 5b) with the average bond distances of 2.861 Å and 2.869 Å, respectively, ranging from 2.488 to 3.126 Å (Table 1). This is consistent with the calculated effective coordination number (ECN) of 8.17 for both two Ba atoms, even through the distortion of the [Ba_Eu_(1)(O,N)_8_] polyhedron was calculated to be nearly two times of that of [Ba_Eu_(2)(O,N)_8_]. The centroid of the coordination polyhedron is at the crystal coordinates (0.5821, 0.7249, 0.1128) and (0.8650, 0.2340, 0.0339) with the polyhedron volume [27] of 97.22 Å^3^ and 98.03 Å^3^ for (Ba/Eu)1 and (Ba/Eu)2, respectively. In the [Ba_Eu_(O,N)_8_] polyhedra, among eight oxygen atoms only two bridging (O, N) atoms are connected with the Ba/Eu atoms, viz., (O/N)3 and (O/N)4 for (Ba/Eu)1 and (O/N)6 and (O/N)8 for (Ba/Eu)2 (Figure 5b). Moreover, the point group symmetry of [Ba_Eu_(1)(O,N)_8_] and [Ba_Eu_(2)(O,N)_8_] is the same with the point symmetry of C1 in the Ba_4-y_Eu_y_Si_6_O_16-3x/2_N_x_ lattice. A combination of similar average bond distances Ba/Eu-(O, N) or the coordination polyhedron volume with the same coordination number (*i.e.*, C.N. = 8) and same point symmetry C1, the characters of the two luminescent centers of Eu^2+^ occupied on the Ba sites could be very similar without distinct difference as expected since the luminescence of Eu^2+^ strongly depend of the local structure surrounded the luminescent centers [28].

### 4.3. Photoluminescence properties

**Figure 6:**
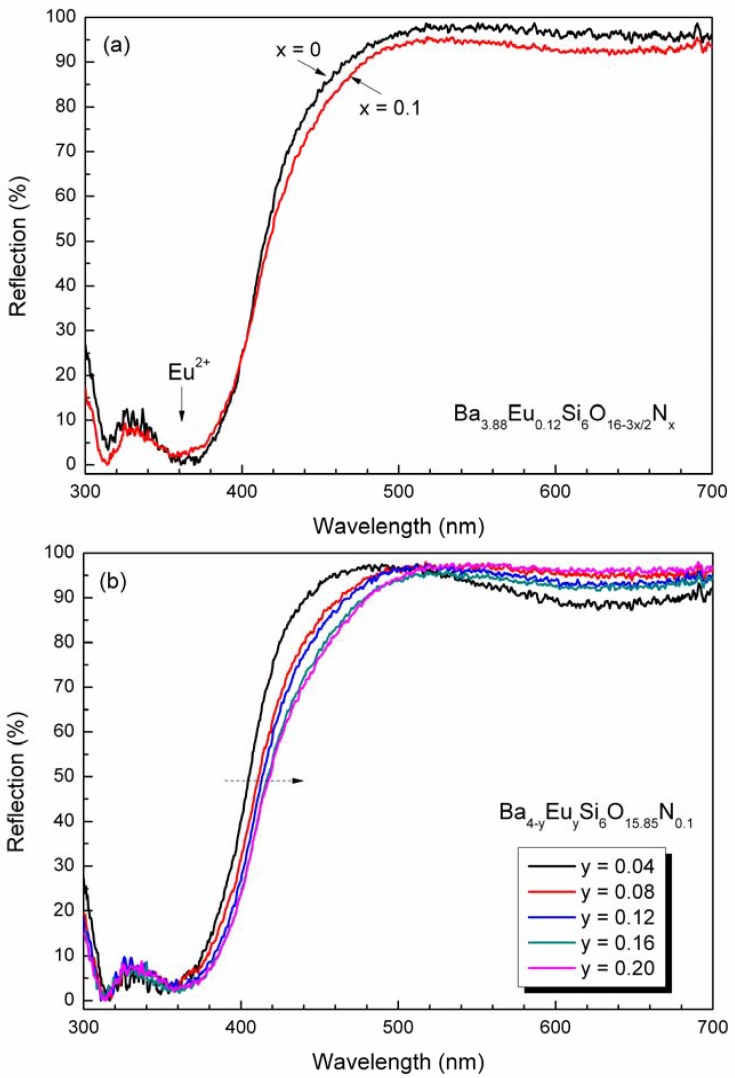
Diffuse reflection spectra of Ba_4-y_Eu_y_Si_6_O_16-3x/2_N_x_ as the function of (a) the nitrogen content (x), and (b) the Eu^2+^ concentration (y).

Figure 6a shows the diffuse reflection spectra of Ba_3.88_Eu_0.12_Si_6_O_16-3x/2_N_x_ of x = 0 and x = 0.1. There is a broad absorption band centered at about 360 nm for the Eu^2+^ concentration of 3 mol % in oxide (x = 0) and oxynitride (x = 0.1) based phosphors, which is associated with the 4f ➔ 5d transition of Eu^2+^. It is clearly seen that the absorption edge of Eu^2+^ shifts to long wavelength (*i.e.*, low energy) from about 443 to 447 nm by introducing nitrogen, suggesting that a small amount of nitrogen indeed can be incorporated into the oxide lattice of Ba_4_Si_6_O_16_ by partial substitution oxygen because nitrogen can narrow the band gap energy of the host through introduction of impurity levels close the bottom of the conduction bands [29]. On the other hand, as shown in Figure 6b, the absorption edge of Ba_4-y_Eu_y_Si_6_O_16-3x/2_N_x_ (x = 0.1) also significantly shifts to longer wavelengths with an increase of the doping Eu^2+^ concentration due to the reabsorption between the Eu^2+^ ions [28].

**Figure 7:**
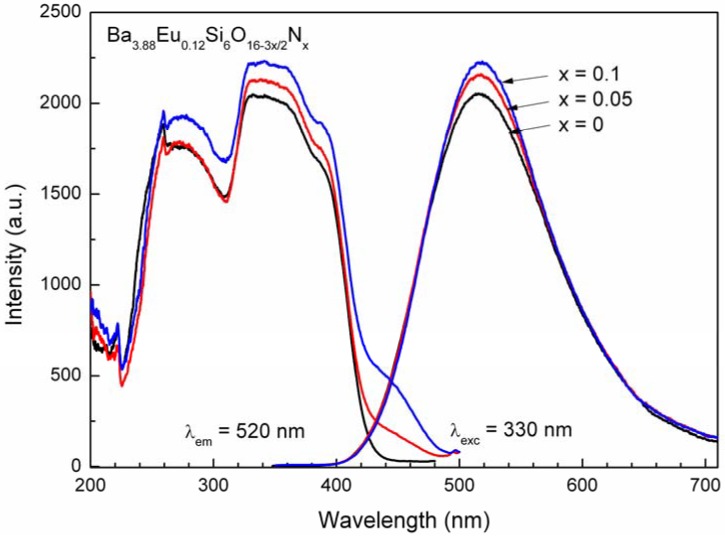
Excitation and emission spectra of Ba_3.88_Eu_0.12_Si_6_O_16-3x/2_N_x_ with the variation of the nitrogen content (x).

Figure 7 represents the excitation and emission spectra of Ba_4-y_Eu_y_Si_6_O_16-3x/2_N_x_ as a function of the doping nitrogen content. The excitation spectra consist of two major bands centered at about 276 and 340 nm accompanying with a strong shoulder at about 386 nm, in fair agreement with the observed absorption band of Eu^2+^ (~365 nm) in the reflection spectra (Figure 6). Corresponding to the incorporation of nitrogen into the lattice, a weak shoulder also appears at about 440 nm, which can be enhanced at high nitrogen content. When excited at UV and UV/blue (*i.e.*, 360*–*400 nm), Ba_3.88_Eu_0.12_Si_6_O_16-3x/2_N_x_ (x = 0*–*0.1) shows bright green emission with a broad emission band peaking at about 520 nm, arising from the 4f^6^5d^1^ ➔ 4f^7^ transition of the Eu^2+^ ion, whose position show a slight red shift (1 ~ 2 nm) in comparison with that of oxide based one (x = 0) due to the presence of nitrogen that results in an increase of covalent bonding in the lattice. It is worth noting that the position of the emission band of Eu^2+^ obtained in this work for x = 0 is significantly different with the studies on Ba_2_Si_3_O_8_:Eu^2+^ [7], where the emission band was found to be located at about 485 nm or 500 nm, giving bluish green light. As mentioned above (see section 4.2), since the two luminescence centers of Eu^2+^ are so similar in structures that the two emission bands of Eu^2+^ can hardly distinguished, namely they are almost completely overlapping together (Figure 7). Additionally, both the excitation and emission intensity of Ba_4-y_Eu_y_Si_6_O_16-3x/2_N_x_ exhibit a slight increased tendency with the nitrogen content increasing in the x range of 0*–*0.1.

**Figure 8:**
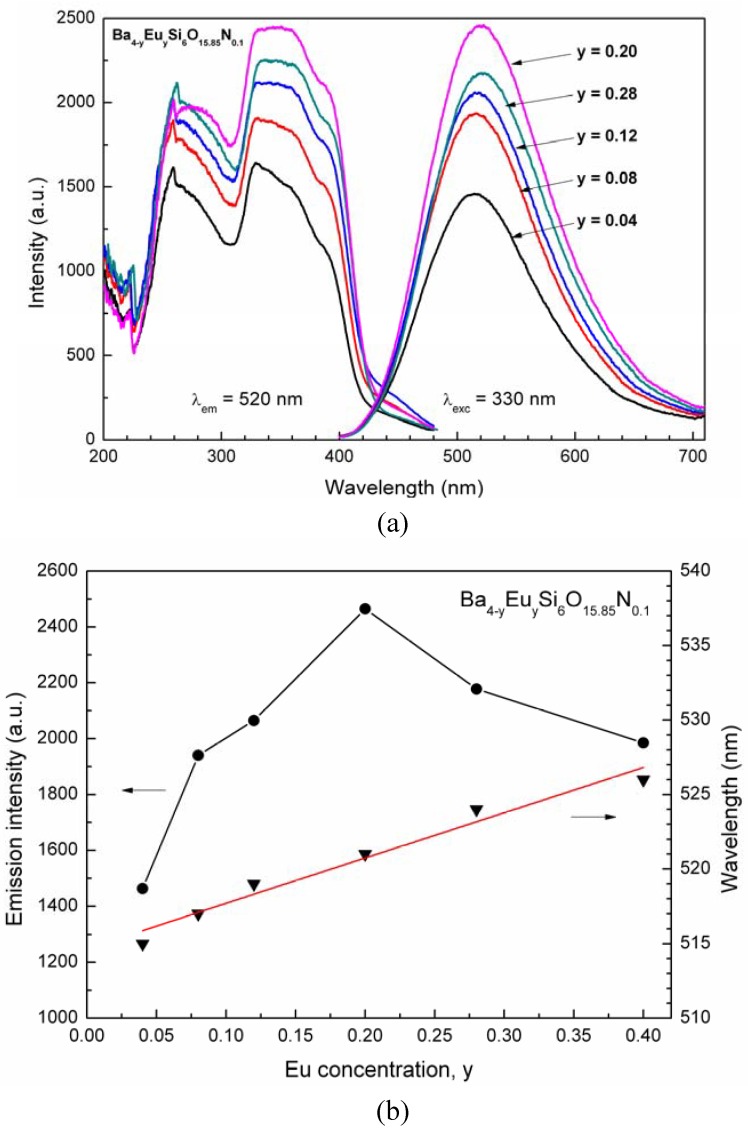
Excitation and emission spectra of Ba_4-y_Eu_y_Si_6_O_15.85_N_0.1_ (a), and the relationships between emission intensity and wavelength with the Eu^2+^ concentration (y).

Figure 8 illustrates the photoluminescence properties of Ba_4-y_Eu_y_Si_6_O_15.85_N_0.1_ as a function of the Eu^2+^ concentration (y). As seen, the excitation and emission intensity of Ba_4-y_Eu_y_Si_6_O_15.85_N_0.1_ increase with an increase of the Eu^2+^ concentration in the range of 0.04 ≤ y < 0.2. The photoluminescence (PL) intensity reaches a maximum value at the Eu^2+^ concentration y ≈ 0.2. While when y surpasses 0.2, due to concentration quenching within the Eu^2+^ ions, the PL intensity shows a decrease tendency (Figure 8b). Similar to the reflection spectra, the right wing of the excitation spectra also shows a slight red shift originated from the reabsorption of Eu^2+^ [28]. As usual, while the position of the emission band of Ba_4-y_Eu_y_Si_6_O_15.85_N_0.1_ linearly shifts to longer wavelengths from 515 to 526 nm (Figure 8b) mainly caused by the energy transfer and/or reabsorption within the Eu^2+^ ions, as well as the increased Stokes shift, for example the Stokes shift is estimated to be 6600 and 6700 cm^-1^ for y = 0.04 and y = 0.2, respectively.

**Figure 9:**
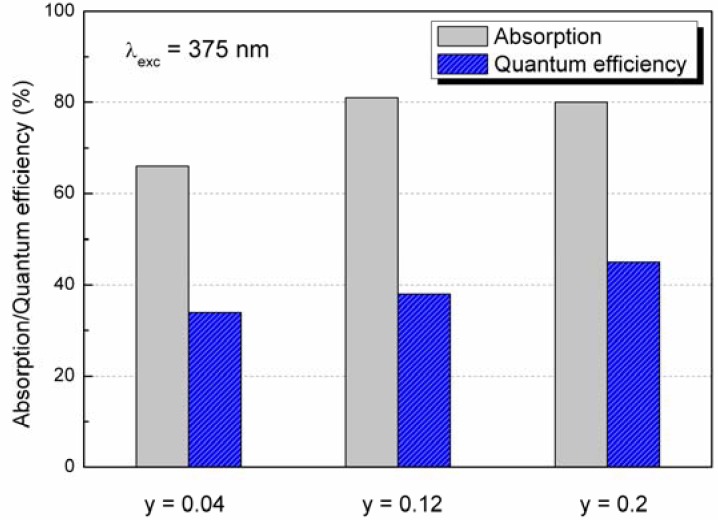
Quantum efficiency of Ba_4-y_Eu_y_Si_6_O_15.85_N_0.1_ at different Eu^2+^ concentrations (y = 0.04, 0.12, 0.2).

The relationships between the absorption and quantum efficiency of Ba_4-y_Eu_y_Si_6_O_15.85_N_0.1_ with the Eu^2+^ concentration are given in Figure 9. The preliminary results showed that the highest absorption and quantum efficiency could be achieved with 80% and 46%, respectively, when y = 0.2 in Ba_4-y_Eu_y_Si_6_O_15.85_N_0.1_ under a monitoring wavelength at 375 nm.

**Figure 10:**
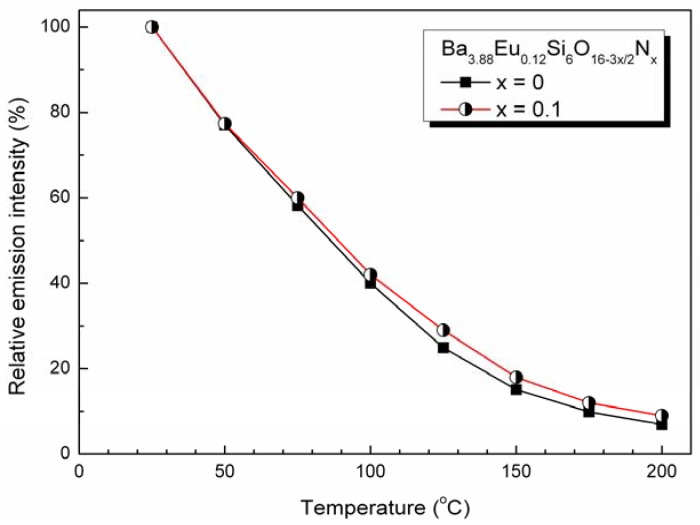
Temperature dependence of Ba_3.88_Eu_0.12_Si_6_O_16-3x/2_N_x_ for x = 0 and 0.1 (λ_exc_ = 340 nm).

The thermal stability of the Ba_3.88_Eu_0.12_Si_6_O_16-3x/2_N_x_ phosphors at high temperature is given in Figure 10. Surprisingly, the thermal quenching rate is very high and the relative emission intensity shows a nearly linear decrease with the temperature rising. The thermal quenching temperature T_1/2_ is at about 100 °C for Ba_4-y_Eu_y_Si_6_O_15.85_N_0.1_ (y = 0.1). This behavior may be mainly related to the crystal structure and composition. As compared to oxide based phosphors (y = 0), with the incorporation of nitrogen, e.g., y = 0.1, the thermal stability of oxynitride based Ba_3.88_Eu_0.12_Si_6_O_16-3x/2_N_x_ phosphor has been slightly improved. The incorporation of nitrogen into Ba_4_Si_6_O_16_:Eu^2+^ can increase the rigidity of the host lattice because Si-(O, N) has high covalent bond than that of Si-O as expected. On the other hand, due to the presence of nitrogen the Eu^2+^ ions are more stable in air at high temperature. Those two reasons may be responsible for an increase of the thermal stability. Furthermore, it has been found that the thermal stability of Ba_4-y_Eu_y_Si_6_O_15.85_N_0.1_ could be further increased by the modification the chemical composition with alkaline earth cation that results will be reported elsewhere. As a whole, with improved temperature dependence, novel oxynitride Ba_4-y_Eu_y_Si_6_O_16-3x/2_N_x_ could be a potential candidate green conversion phosphor for application in white-light UV-LEDs.

## 5. Conclusions

Novel Ba_4-y_Eu_y_Si_6_O_16-3x/2_N_x_ phosphor has been synthesized by a solid state reaction at 1200*–*1300 °C in a N_2_-H_2_ (5%) atmosphere. First-principles calculations indicate that the host of Ba_4_Si_6_O_16_ is an insulator with a direct energy gap of 4.6 eV. The upper valence bands are determined by O-2p states hybridized with Ba-6s and Si-3p states and the conduction bands are dominated by Ba-6s states. The maximum solubility of nitrogen was achieved at about x = 0.1 in Ba_3.88_Eu_0.12_Si_6_O_16-3x/2_N_x_ (y = 0.12), which has the lattice parameters *a* = 12.4663(5) Å, *b* = 4.6829(2) Å, *c* = 13.9236(6) Å, and *β* = 93.61(1)° in monoclinic system (space group *P*2_1_/c, Z = 2). When excitation under UV to 400 nm, Eu^2+^-activated Ba_4_Si_6_O_15.85_N_0.1_ emits green light with a broad emission band peaking at about 520 nm associated with the transition of 4f^6^5d^1^ ➔ 4f^7^ of Eu^2+^, and the position of the emission bands can be modified by varying the Eu^2+^ concentration. The quantum efficiency of Ba_4-y_Eu_y_Si_6_O_15.85_N_0.1_ is about 46% for y = 0.2 under excitation wavelength at 375 nm. The thermal quenching temperature is about 100 °C. With improved thermal stability oxynitride based Ba_4-y_Eu_y_Si_6_O_16-3x/2_N_x_ phosphor is promising white UV-LED applications.
